# The impact of evodiamine on human anaplastic thyroid cancer therapy—an *in vitro* and *in vivo* study

**DOI:** 10.7150/ijms.122604

**Published:** 2025-10-27

**Authors:** Yin-Che Lu, Tsung-Hsing Lin, Kai-Liang Tang, Chin-Ho Kuo, Yi-Sheng Zhang, Yi-Ping Chang, Shu-Hsin Chen, Yi-Zhen Li, Pei-Wen Zhao, Jen-Hsien Lin, Ying-Ray Lee

**Affiliations:** 1 Division of Hematology-Oncology, Ditmanson Medical Foundation Chia-Yi Christian Hospital, Chiayi, Taiwan.; 2 Department of Emergency Medicine, Kuang Tien General Hospital, Taichung 433, Taiwan.; 3 Taichung District Agricultural Research and Extension station, Ministry of Agriculture, Changhua County, Taiwan.; 4 Department of Medical Research, Ditmanson Medical Foundation Chiayi Christian Hospital, Chiayi, Taiwan.; 5 Bone and Joint Research Center, Chang Gung Memorial Hospital, Taoyuan, Taiwan.; 6 School of Medicine, College of Medicine, Kaohsiung Medical University, Kaohsiung, Taiwan.; 7 Department of Microbiology and Immunology, College of Medicine, Kaohsiung Medical University, Kaohsiung, Taiwan.; 8 Master of Science Program in Tropical Medicine, College of Medicine, Kaohsiung Medical University, Kaohsiung, Taiwan.; 9 Faculty of Post-Baccalaureate Medicine, College of Medicine, Kaohsiung Medical University, Kaohsiung, Taiwan.; 10 Center for Tropical Medicine and Infectious Disease, Kaohsiung Medical University, Kaohsiung, Taiwan.; 11 Department of Medical Research, Kaohsiung Medical University Hospital, Kaohsiung, Taiwan.

**Keywords:** thyroid cancer, evodiamine, anticancer activity, apoptosis

## Abstract

Thyroid cancer (TC) is the most common endocrine malignancy, with anaplastic thyroid cancer (ATC) being the most aggressive subtype. Evodiamine (EVO), a bioactive compound derived from *Evodia rutaecarpa*, possesses anti-inflammatory and anti-tumor properties, though its effects on ATC remain underexplored. This study investigated the anticancer potential of EVO using ARO and SW579 ATC cell lines in both *in vitro* and *in vivo* models. EVO significantly inhibited cell proliferation, induced G2/M phase arrest, and increased the sub-G1 population, indicating growth inhibition and cell death. Mechanistically, EVO activated the intrinsic caspase-dependent apoptotic pathway and triggered autophagy, as shown by autophagosome accumulation and elevated LC3-II levels. Importantly, blocking autophagy attenuated caspase activation, suggesting that autophagy contributes to EVO-induced apoptosis. Moreover, oral EVO administration markedly suppressed tumor growth in a nude mouse xenograft model without causing liver or kidney toxicity. TUNEL assay further confirmed enhanced tumor cell apoptosis *in vivo*. These results highlight EVO as a promising therapeutic candidate for ATC by simultaneously activating autophagy and apoptosis pathways.

## Introduction

Thyroid cancer (TC) is the most common malignancy of the endocrine system, with a steadily rising global incidence 1, 2. It is about twice as common in women as in men. Major risk factors include increasing age, a family history of thyroid disease, and exposure to radiation during childhood 3, 4. According to histopathology, TC arises from two major cell types: follicular epithelial cells and parafollicular neuroendocrine (C) cells. Tumors originating from follicular cells include papillary thyroid carcinoma (PTC), follicular thyroid carcinoma (FTC), poorly differentiated thyroid carcinoma (PDTC), and anaplastic thyroid carcinoma (ATC). PTC and FTC are collectively known as differentiated thyroid cancers (DTC), while ATC is an undifferentiated form. Tumors from C cells are classified as medullary thyroid carcinoma (MTC) 5, 6.

Among these, PTC is the most prevalent, accounting for approximately 70-75% of cases, followed by FTC at around 10-15% 6, 7. FTC tends to metastasize through the bloodstream, particularly to bones and lungs, occurring in 7-23% of cases, and the long-term survival ranges of metastatic FTC is 31-43% 8, 9. While patients with DTC generally have an excellent prognosis when treated with total thyroidectomy, lymph node dissection, and radioactive iodine therapy, those with recurrent or metastatic disease face limited treatment options. The 10-year survival rate for such patients falls below 10% 10, 11. ATC, although rare (2-5% of TC), is highly aggressive and one of the most lethal human malignancies, with an average survival time of only 3 to 9 months 12. It responds poorly to both chemotherapy and radiotherapy, as do recurrent or metastatic PTC and FTC 13, 14.

In addition to conventional therapies, novel molecularly targeted agents and immunotherapies represent promising emerging treatment options. Targeted therapies such as Lenvatinib (multi-target TKI), Sorafenib (VEGFR and Raf inhibitor), Vemurafenib and Dabrafenib (BRAF inhibitors), Trametinib (MEK inhibitor), and Selpercatinib/Pralsetinib (RET inhibitors), as well as immune checkpoint inhibitors like Pembrolizumab and Nivolumab, have been reported to improve therapeutic outcomes in human thyroid cancers 15. However, the high cost of these agents often places a substantial economic burden on patients during long-term treatment. Moreover, despite initial efficacy, resistance to targeted therapies frequently develops, ultimately leading to disease progression. Therefore, there is an urgent need to develop more effective therapies targeting advanced or treatment-resistant thyroid cancers.

Evodiamine (EVO) is a natural alkaloid extracted from *Evodia rutaecarpa*, widely studied for its diverse pharmacological properties 16. Recent research has highlighted its anti-inflammatory, neuroprotective, and notably, anti-tumor activities 17. EVO exerts anti-cancer effects by inhibiting tumor cell proliferation, migration, and invasion, while inducing apoptosis and cell cycle arrest through pathways involving reactive oxygen species (ROS), caspase activation, and MAPK signaling 18, 19. It has demonstrated efficacy against various human cancers, including esophageal squamous carcinoma, lung cancer, breast cancer, hepatoma, colon cancer, prostate cancer, bladder cancer, pancreatic cancer, gastric cancer, ovarian cancer, melanoma, glioblastoma, T-lymphoma and thyroid carcinoma 20-22. In thyroid cancer, EVO suppresses the growth of both PTC and ATC cancer cells and induces cellular apoptosis, as well as enhances the effect of histone deacetylase inhibitors 23-26. Our previous study also demonstrates the effects of EVO on FTC 10. Moreover, novel EVO-based theranostic agents have been developed by linking fluorophores and tumor-targeting units to improve drug delivery and visualization in hypoxic tumors 27. Beyond oncology, EVO also shows potential in neurological diseases; it promotes remyelination by inhibiting NLRP3 inflammasome-mediated pyroptosis in microglia, as shown in cuprizone- and EAE-induced demyelination models 28. Despite extensive research, the application of EVO on human thyroid cancers remains unclear. Continued investigation is needed to explore its therapeutic potential *in vivo* in refractory thyroid cancers.

## Materials and Methods

### Cell culture

The human anaplastic thyroid cancer cell line ARO was generously provided by Dr. Chih-Yuan Wang 29, while the SW579 cell line was obtained from the Bioresource Collection and Research Center (BCRC), Taiwan. ARO cells were cultured in RPMI-1640 medium (Gibco BRL, Grand Island, NY, USA) supplemented with 10% fetal bovine serum (FBS), 100 U/mL penicillin, and 0.1 μg/mL streptomycin, maintained at 37°C in a humidified incubator with 5% CO₂. In contrast, SW579 cells were maintained in 90% Leibovitz's L-15 medium (Gibco BRL) supplemented with 10% FBS, 100 U/mL penicillin, and 0.1 μg/mL streptomycin at 37°C in a CO₂-free humidified environment.

### Cell viability assay

Cells (5 × 10³ cells per well) were seeded into 96-well plates and allowed to adhere overnight. The following day, cells were treated with either control medium containing 0.01% dimethyl sulfoxide (DMSO; Sigma-Aldrich, St. Louis, MO, USA) or medium supplemented with evodiamine (Sigma-Aldrich) at the indicated concentrations and time points. Cell viability was assessed using the Cell Counting Kit-8 (CCK-8; Enzo Life Sciences, Farmingdale, NY, USA) according to the manufacturer's instructions. All experiments were performed in triplicate, and data were obtained from three independent experiments.

### Colony formation assay

Cells were seeded in 6-well plates at a density of 1 × 10³ cells per well and maintained in complete growth medium at 37°C. After overnight incubation to allow cell attachment, the medium was replaced with either control medium containing 0.01% DMSO or medium supplemented with EVO. Cells were then incubated for 10 days, with medium changes every 2-3 days. At the end of the incubation period, colonies were fixed and stained with 10% crystal violet solution (Sigma-Aldrich). Colony number and size were subsequently assessed and recorded under a microscope.

### Cell cycle analysis

Cells were treated with EVO at concentrations of 1 μM or 2.5 μM, or with 0.01% DMSO as a control, for 48 and 72 hours. Following treatment, cells were harvested and fixed in 70% ethanol at 4°C overnight. After twice washes with PBS, the cells were resuspended in 500 μL of propidium iodide (PI) staining buffer (Sigma-Aldrich) and incubated in the dark at room temperature for 30 minutes. DNA content was then analyzed using a FACScan flow cytometer (Becton Dickinson, San Diego, CA, USA) and ModFit LT 3.3 software.

### Cellular apoptosis analysis

Cells were treated with various concentrations of EVO or 0.01% DMSO as a control for the indicated durations. After treatment, cells were washed twice with PBS and centrifuged at 1500 × g for 10 minutes to remove residual medium. The cell pellets were resuspended in 100 μL of binding buffer containing 2 μL Annexin V-FITC and 2 μL propidium iodide (PI) (Sigma-Aldrich), followed by incubation at room temperature in the dark for 15 minutes. Apoptotic cells were then analyzed by flow cytometry using a FACScan instrument (Becton Dickinson). A total of 1 × 10⁵ cells were evaluated per sample. All experiments were performed independently in triplicate.

### Western blotting

To investigate the molecular mechanisms underlying EVO-induced cell cycle arrest and apoptosis, cells were treated with either control medium (containing 0.01% DMSO) or EVO at the indicated concentrations. After treatment, total cellular proteins were extracted and quantified. Equal amounts of protein were separated by SDS-polyacrylamide gel electrophoresis (SDS-PAGE) and subsequently transferred onto polyvinylidene difluoride (PVDF) membranes. After blocking with 5% non-fat milk, membranes were incubated with specific primary antibodies followed by appropriate horseradish peroxidase (HRP)-conjugated secondary antibodies. Protein bands were visualized using enhanced chemiluminescence (ECL), following the procedure described in our previous studies 30-33. The primary antibodies used in this study included: cdc2 (#9116), cdc25C (#4688), p21 (#2947), caspase-3 (#9662), caspase-9 (#9502), and PARP (#9542) (from Cell Signaling Technology, Danvers, MA, USA), and cyclin A (GTX27956), cyclin B1 (GTX100911), and GAPDH (GTX100118) (from GeneTex, Hsinchu City, Taiwan).

To confirm the involvement of caspase activation in EVO-induced apoptosis, cells were pre-treated with the pan-caspase inhibitor Z-VAD-FMK (BioVision, Mountain View, CA, USA). Apoptosis was subsequently assessed by Western blot.

### DNA fragmentation analysis

DNA fragmentation is a hallmark of apoptosis and was evaluated to determine whether EVO induces apoptotic cell death in ATC cells. Cells (1 × 10⁶) were cultured and treated with either control medium containing 0.01% DMSO or EVO for 48 and 72 hours. Following treatment, genomic DNA was extracted using standard procedures. The isolated DNA was subjected to electrophoresis on a 2% agarose gel containing 0.1 μg/mL ethidium bromide (Sigma-Aldrich) and visualized under UV illumination. The presence of characteristic DNA laddering patterns was used as an indicator of apoptosis.

### Autophagosome detection

Cells were seeded onto coverslips placed in 10 cm culture dishes and incubated overnight at 37°C. Following treatment with medium containing either DMSO or evodiamine for 72 hours, autophagosomes were visualized using immunofluorescence staining. Cells were incubated overnight at 4°C with an anti-LC3 primary antibody (Medical and Biological Laboratories, Tokyo, Japan), followed by staining with a fluorescein isothiocyanate (FITC)-conjugated secondary antibody (GeneTex). Nuclei were counterstained with 4',6-diamidino-2-phenylindole (DAPI; Sigma-Aldrich). The presence of autophagosomes was assessed using a laser scanning confocal microscope (LSM800, ZEISS, Oberkochen, Germany).

### In vivo anticancer assay using xenograft nude mouse model

Six-week-old male BALB/cAnN.Cg-*Foxn1 ^nu^*/CrlNarl nude mice were obtained from the National Laboratory Animal Center and acclimatized at the Animal Facility of National Chiayi University, Chiayi, Taiwan. For the in vivo study, the mice were randomized into three groups (n = 7 per group), and each mouse received a s.c. injection of 2 × 10⁶ ARO cells. After 7 days, the control group received oral administration of DMSO, while the treatment groups received oral doses of evodiamine at 5 mg/kg or 15 mg/kg starting as Day 0. Drug administration given once every day.

Tumor volume was measured every 3 days using calipers, and calculated using the formula: (L × S²)/2, where L is the longest diameter and S is the shortest diameter. On Day 21, all mice were euthanized, and tumors were excised and weighed. All animal procedures were performed in compliance with Taiwan's Animal Protection Act and was approved by the Laboratory Animal Care and Use Committee of the National Chiayi University (IACUC Approval No. 101020).

### Statistical analysis

All data are expressed as the mean ± standard deviation (SD) from the indicated number of independent experiments. Statistical analyses were performed using SPSS version 16.0. For datasets with sample sizes greater than 30, comparisons between groups were conducted using Student's *t*-test. For sample sizes fewer than 30, the non-parametric Mann-Whitney *U* test was applied. A *p*-value less than 0.05 was considered statistically significant in all analyses.

## Results

### Evodiamine suppresses cell proliferation in human anaplastic thyroid cancer cells

**Figure 1:**
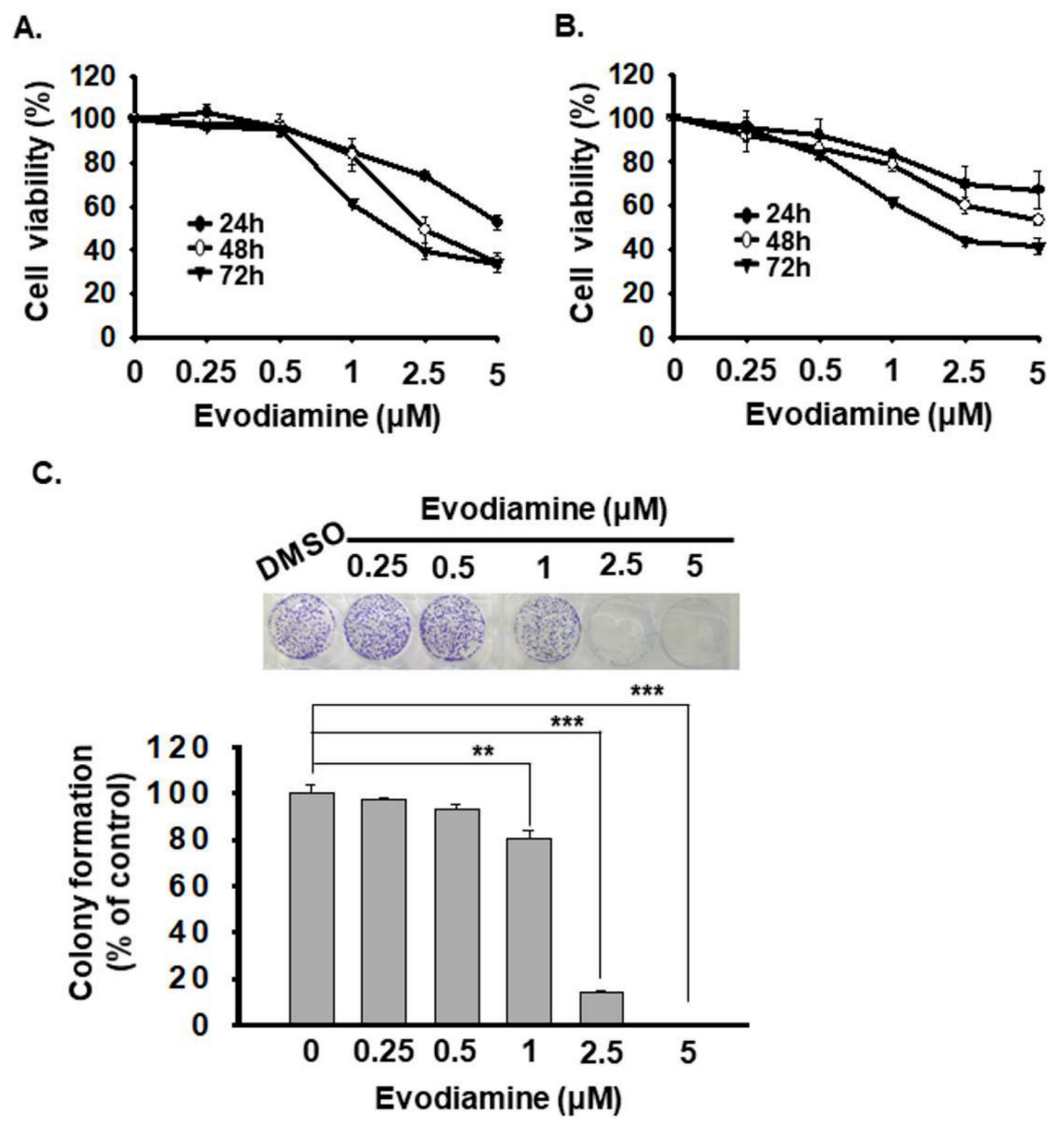
Evodiamine inhibits cell proliferation of human anaplastic thyroid carcinoma cells. (A) ARO and (B) SW579 cells were treated with evodiamine, and the cell viability was evaluated with CCK-8 assay. The results are shown as the mean ± S.D. (n = 6) of three independent experiments. (C) ARO cells were administrated with evodiamine or DMSO, and the colony formation was determined with crystal violet staining after 10 days treatment. DMSO was used as negative control. * comparing with the control group. ** p < 0.01. *** p < 0.001.

To evaluate the anticancer effects of Evodiamine (EVO) on anaplastic thyroid cancer (ATC) cells, we assessed cell viability using the CCK-8 assay in two human ATC cell lines (ARO and SW579). Cells were treated with various concentrations of EVO (0, 0.25, 0.5, 1, 2.5, and 5 μM) for 24, 48, and 72 hours. The results indicated that EVO inhibited cell proliferation in a dose- and time-dependent manner in both cell lines (Figure 1A and 1B). The corresponding IC50 values are presented in Table 1. Among the tested cell lines, ARO cells exhibited higher sensitivity to EVO treatment than SW579 cells.

Furthermore, to evaluate the effect of EVO on long-term proliferative capacity, we conducted a colony formation assay. However, SW579 cells exhibited poor colony-forming ability. In contrast, EVO significantly reduced ARO cell colony formation in a concentration-dependent manner (Figure 1C).

Taken together, these findings support EVO as a promising anticancer agent for targeting ATC cells. Based on its ability to reduce the viability of both ARO and SW579 cells to below 50% of the control, 2.5 μM was selected as an effective dose for subsequent experiments.

### Evodiamine administration causes G2/M cell cycle arrest and elevates sub-G1 in human anaplastic thyroid cancer cells

**Figure 2:**
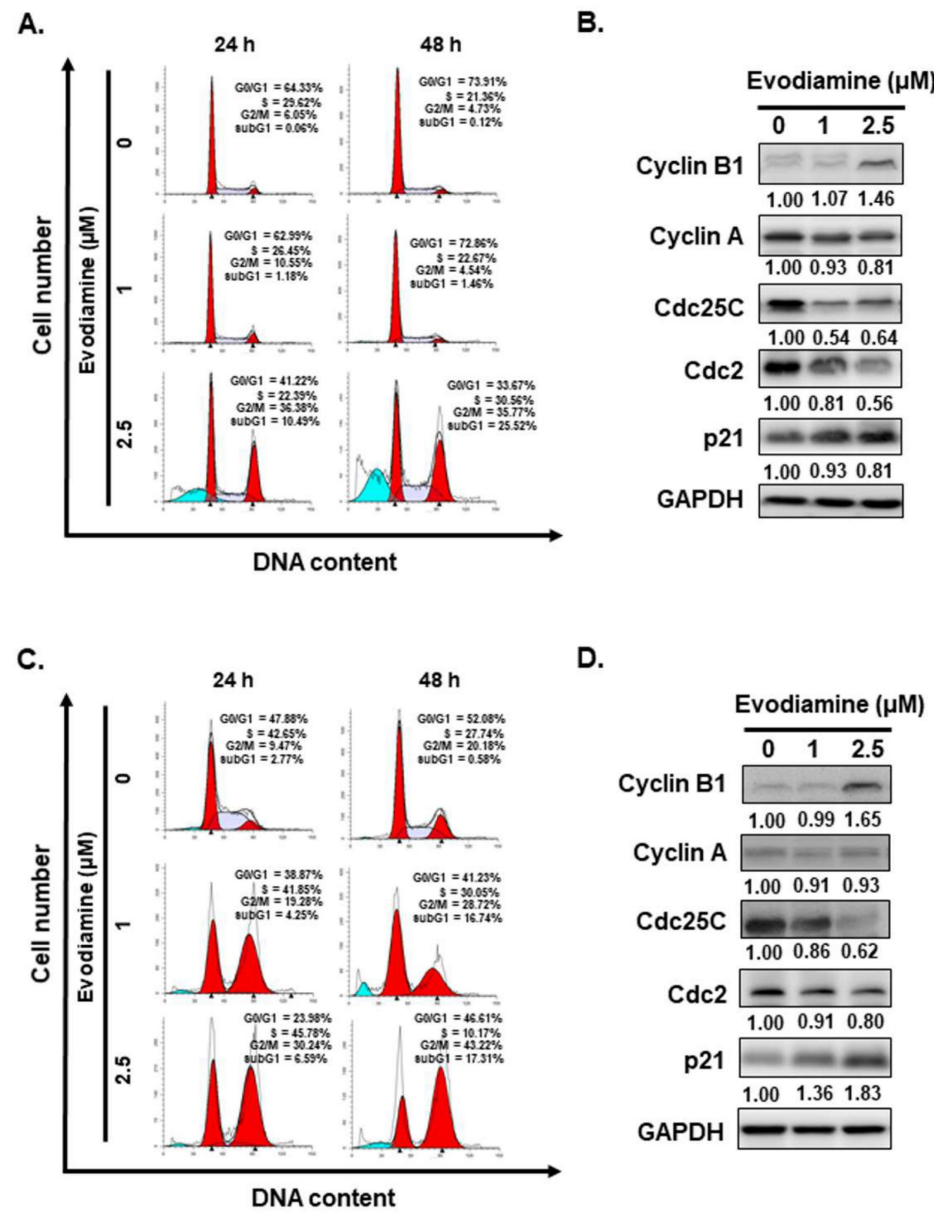
Evodiamine induces cell cycle arrest and sub-G1 population increased in human anaplastic thyroid carcinoma cells. (A) ARO and (C) SW579 cells were incubated with evodiamine, and the cell cycle was examined with flow cytometry. Moreover, the sub-G1 population in (A) ARO and (C) SW579 cells was determined with flow cytometry. The cell cycle markers were examined by western blotting in (B) ARO and (D) SW579 cells treated with evodiamine. DMSO was used as negative control. Three independent experiments each for western blotting and flow cytometry were conducted, and a representative data was shown.

Given that EVO treatment significantly reduced the viability of ATC cells (Figure 1A and 1B), subsequent investigations were conducted to determine whether this growth inhibition was associated with alterations in cell cycle progression and the induction of cell death. Flow cytometric analysis of cell cycle distribution revealed a substantial accumulation of cells in the G2/M phase following 24 to 48 hours of EVO exposure across all examined ATC cell lines (Figure 2A and 2C). suggesting that EVO induces cell cycle arrest at the G2/M checkpoint.

To further elucidate the mechanisms underlying EVO-induced cell cycle arrest, the expression levels of key cell cycle regulatory proteins, including cyclin A, cyclin B1, cdc2, cdc25C, and p21, were examined by Western blot analysis. As shown in Figure 2B and 2D, EVO treatment led to a decrease in cyclin A, cdc2 and cdc25C expression, accompanied by an upregulation of cyclin B1 and p21, supporting the notion that EVO induces G2/M phase arrest in both ARO and SW579 cells.

Interestingly, flow cytometric analysis revealed a marked increase in the sub-G1 population in ARO and SW579 cells following EVO treatment (Figure 2A and 2C), suggesting the occurrence of cell death. Based on this observation, we further investigated whether EVO induces apoptosis in ARO and SW579 cells.

### Evodiamine induces cell apoptosis through intrinsic caspase-dependent pathway in human anaplastic thyroid cancers

To evaluate whether the induction of cellular apoptosis contributes to the anticancer activity of evodiamine (EVO) in anaplastic thyroid cancer (ATC) cells, flow cytometry, Western blotting, and DNA fragmentation assays were performed on ARO and SW579 cells following EVO treatment.

**Figure 3:**
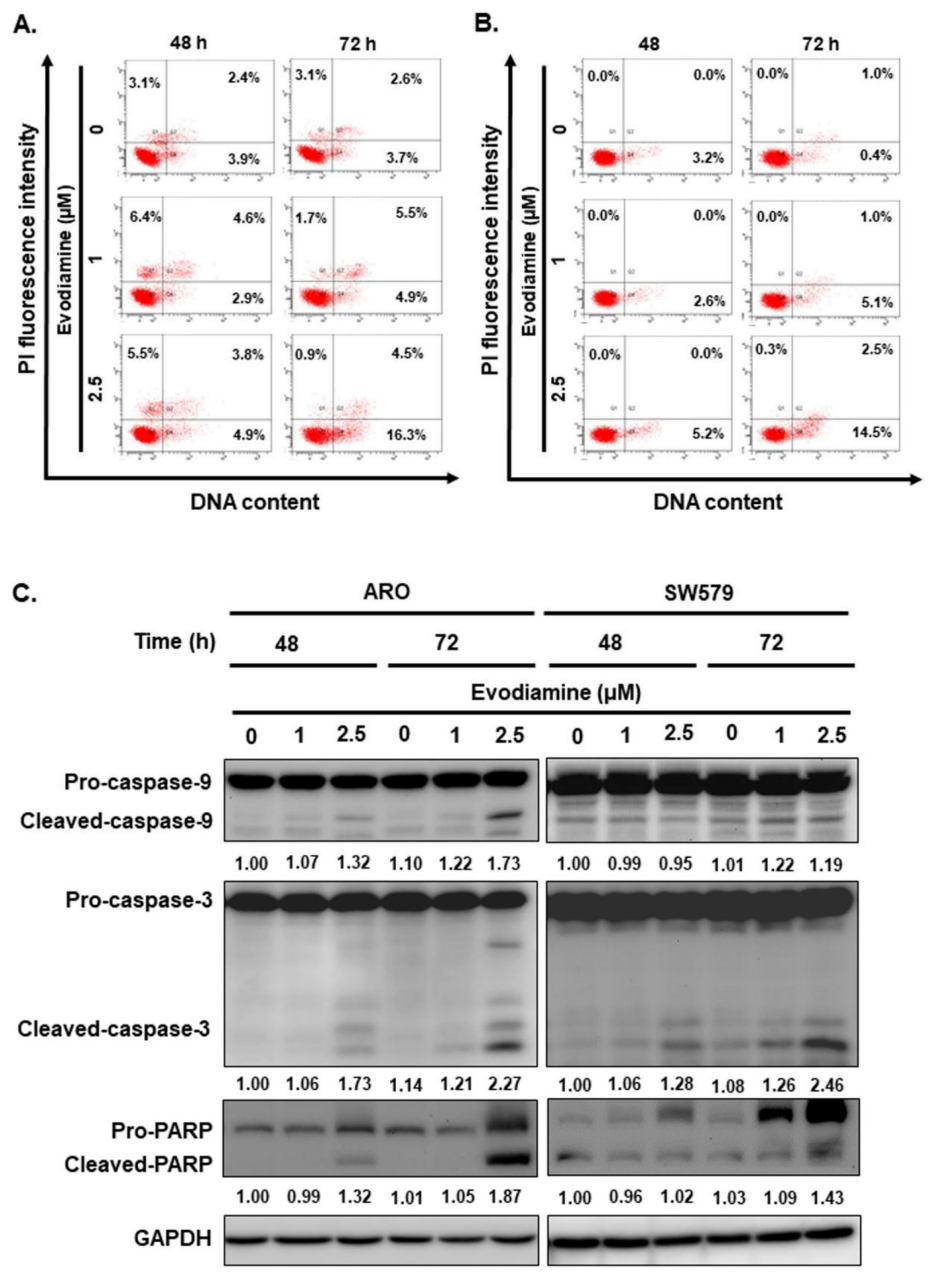

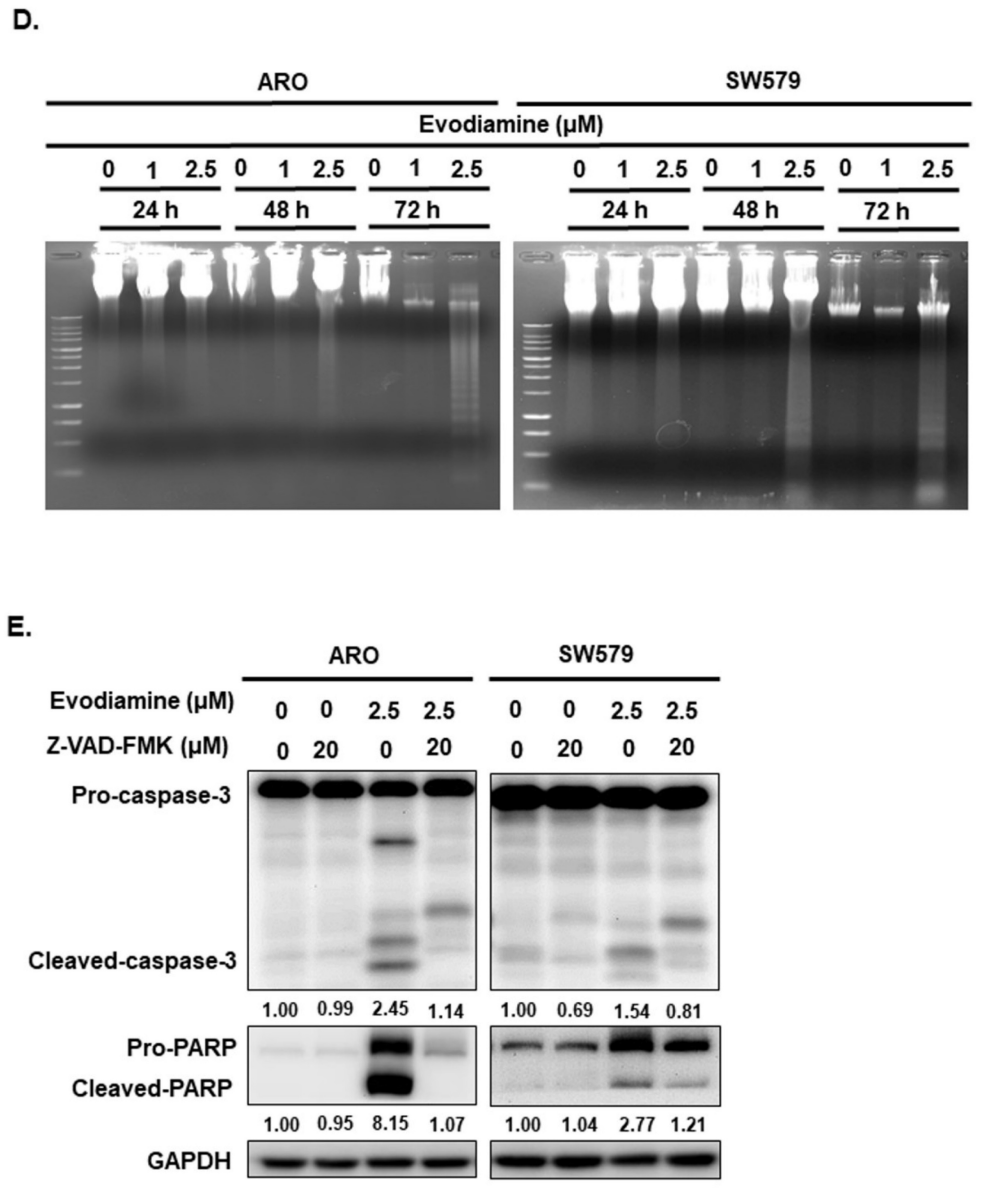
Evodiamine induces apoptosis in human anaplastic thyroid carcinoma cells. (A) ARO and (B) SW579 cells were incubated with evodiamine, and the apoptotic cells were determined with flow cytometry. (C) The expressions of caspase-3, -9, and PARP protein were assessed using western blotting in cells treated with evodiamine. (D) DNA fragmentation was detected in the cells under evodiamine treatment. (E) Confirmation evodiamine mediated apoptosis was via caspase-dependent pathway in ARO and SW579 cells and determined with western blotting after 72 h treatment. DMSO was used as negative control. Three independent experiments each were conducted for flow cytometry and western blotting; Western blots and flow cytometry dot plots of a representative experiment were shown. * compared with the control group.

EVO induced apoptosis in a dose-dependent manner in both cell lines, as determined by Annexin V/PI staining (Figure 3A and 3B). To further elucidate the underlying apoptotic mechanisms, we examined the expression and cleavage of key apoptotic markers, including caspase-8, caspase-9, caspase-3, and poly (ADP-ribose) polymerase (PARP). The results demonstrated that caspase-9, caspase-3, and PARP were significantly activated upon EVO treatment (Figure 3C), while caspase-8 levels remained unchanged (data not shown), suggesting that EVO-induced apoptosis is primarily mediated through the intrinsic (mitochondrial) pathway. To substantiate these observations, a DNA fragmentation assay was conducted, confirming the presence of apoptosis following EVO exposure (Figure 3D).

To further validate the role of caspases in EVO-induced apoptosis, ARO and SW579 cells were pretreated with the pan-caspase inhibitor Z-VAD-FMK prior to EVO administration. As shown in Figure 3E, EVO treatment led to robust activation of caspase-3 and cleavage of PARP, indicating active apoptotic signaling. This activation was significantly suppressed in cells pretreated with Z-VAD-FMK, confirming the caspase-dependent nature of EVO-mediated apoptosis.

Collectively, these findings demonstrate that evodiamine induces apoptosis in human ATC cells predominantly via a caspase-dependent intrinsic pathway, supporting its potential as a promising therapeutic agent for anaplastic thyroid cancer.

### Evodiamine induces cell autophagy through intrinsic caspase-dependent pathway in human anaplastic thyroid cancers

Autophagy is a cellular self-degradation mechanism that involves the sequestration of damaged proteins, organelles, or invading pathogens (such as viruses) into autophagosomes, which subsequently fuse with lysosomes for degradation. This process plays a critical role in maintaining cellular homeostasis and energy balance. In the context of cancer, autophagy exhibits a dual role, functioning as both a tumor suppressor and a tumor promoter at different stages of tumor development. Consequently, autophagy has garnered attention as a potential target for anticancer therapy 9, 34-41.

**Figure 4:**
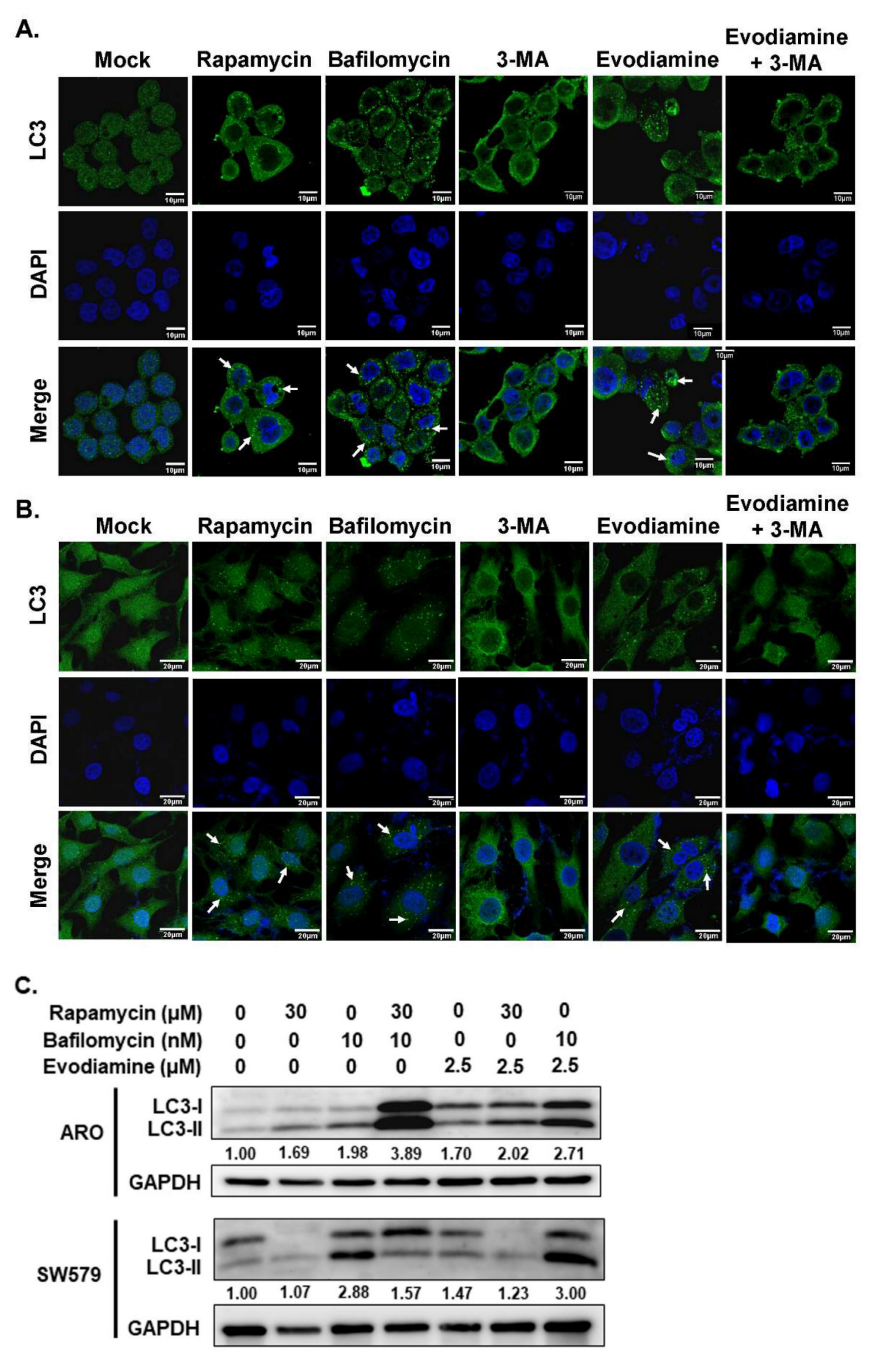
Evodiamine induces autophagy in human anaplastic thyroid carcinoma cells. (A) ARO and (B) SW579 cells were treated with evodiamine, and the autophagosome was assessed with immunofluorescence staining after 72 h post-treatment. (C) The expressions of LC3-I and LC3-II were assessed using western blotting in cells treated with evodiamine for 72 h. DMSO was used as negative control. Rapamycin was used as the positive control of autophagy. Bafilomycin was a blocker to suppress autophagic flux. Three independent experiments were conducted for western blotting, and a representative experiment was shown. Arrow was indicated the LC3 puncta as the autophagosome.

In the present study, the modulation of autophagy in anaplastic thyroid cancer (ATC) cells following evodiamine (EVO) treatment was investigated using immunofluorescence staining and Western blot analysis. As shown in Figures 4A and 4B, autophagosome formation was markedly increased following treatment with rapamycin, a known autophagy inducer, and bafilomycin, an inhibitor of autophagic flux. In contrast, treatment with 3-methyladenine (3-MA), an autophagy inhibitor, significantly reduced autophagosome formation. Notably, EVO treatment induced autophagosome accumulation in both ARO and SW579 cells, an effect that was attenuated by co-treatment with 3-MA. These findings suggest that EVO promotes autophagosome formation in ATC cells, likely through the induction of autophagy.

To confirm autophagy induction in ATC cells following EVO treatment, the expression level of LC3-II, a marker of autophagosome formation, was assessed by Western blot analysis. As shown in Figure 4C, LC3-II accumulation was observed in cells treated with rapamycin, a known autophagy inducer, as well as in cells treated with bafilomycin, an inhibitor of autophagic flux. Similarly, EVO treatment led to increased LC3-II levels in both ARO and SW579 cells. Notably, combined treatment with EVO and bafilomycin resulted in a further elevation of LC3-II compared to either treatment alone. These results indicate that the accumulation of autophagosomes induced by EVO is primarily due to autophagy induction rather than impaired autophagic flux.

**Figure 5:**
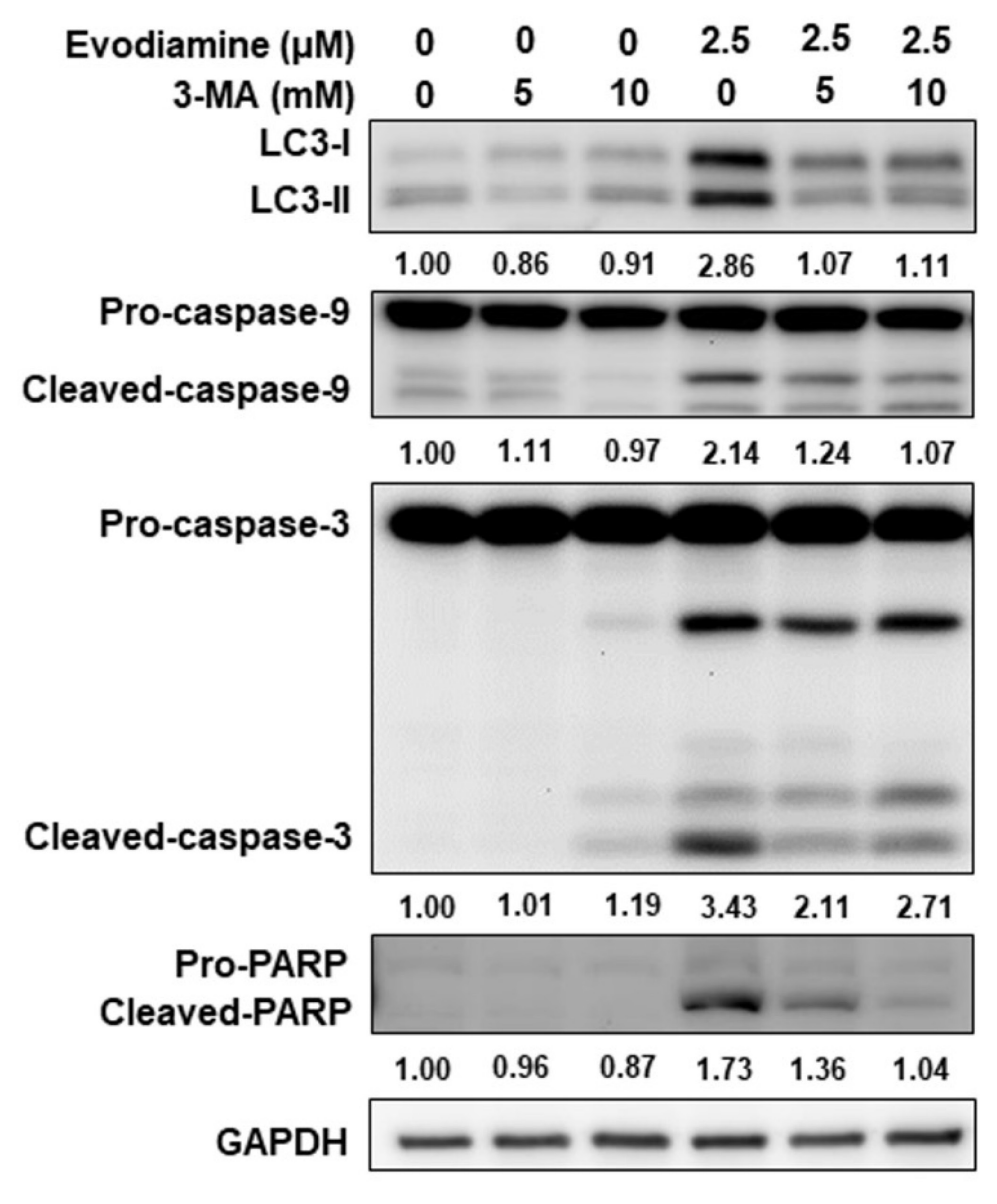
Evodiamine regulated apoptosis was through autophagy modulation. ARO cells were treated with evodiamine for 72 h, and the expression of LC3, caspase-3 and PARP were assessed using western blotting. DMSO was used as negative control. 3-MA was the autophagy inhibitor. Three independent experiments were conducted for western blotting, a representative experiment was shown.

To further confirm autophagy induction by EVO in ATC cells, 3-methyladenine (3-MA), a classical autophagy inhibitor, was employed to block autophagy activation during EVO treatment. As shown in Figure 5, EVO treatment elevated LC3-II expression in both ARO and SW579 cells, whereas co-treatment with 3-MA suppressed this effect, supporting that EVO indeed activates autophagy in ATC cells. Notably, inhibition of EVO-induced autophagy by 3-MA also attenuated EVO-induced caspase activation and the cleavage of PARP (Figure 5), indicating that autophagy may act upstream of apoptosis in EVO-treated ATC cells.

Together, these findings demonstrate that evodiamine induces autophagy in ATC cells, as evidenced by increased autophagosome formation and LC3-II accumulation. Moreover, the attenuation of caspase activation upon autophagy inhibition suggests that EVO-induced autophagy may play a pro-apoptotic role and function upstream of apoptosis in ATC cells.

### Evodiamine reduces tumor growth and induces tumor cell apoptosis *in vivo*

**Figure 6:**
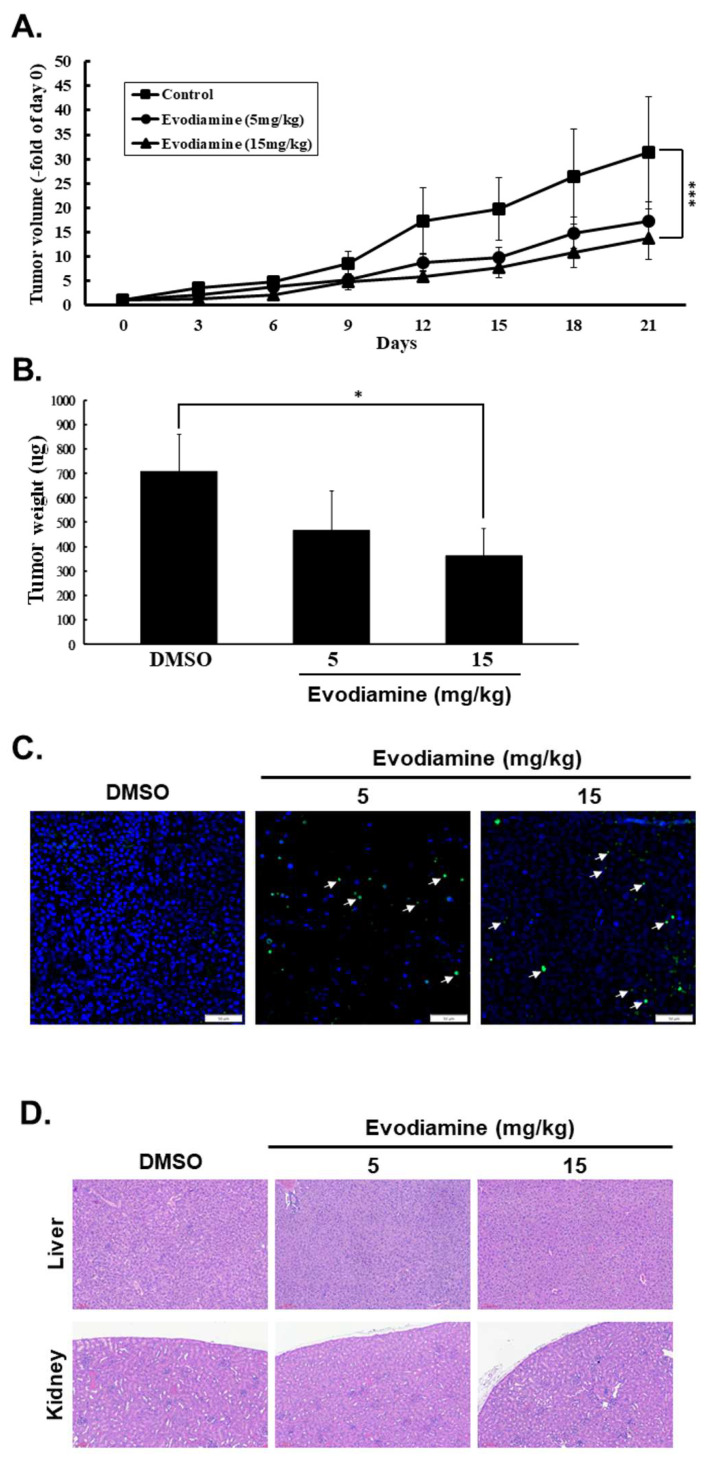
Evodiamine inhibited tumor progression in nude mice. The mice were injected subcutaneously with ARO cells, and the mice were treated with or without evodiamine as described. (A) Subcutaneous tumor volumes in different treatment groups (n=7/group) were detected of days as described. (B) Inhibitory effects of evodiamine on xenograft tumor weight. (C) Apoptosis examination in tumor sections with TUNEL staining. (D) Haematoxylin and eosin staining in liver and kidney specimens of the mice. DMSO was used as negative control. Arrow was indicated the colocalization of apoptosis-induced nuclear DNA fragmentation. * comparing with the control group. * p < 0.05. *** p < 0.001.

EVO has demonstrated potent antitumor activity in various human cancers 20-26. Although EVO against human ATC cells has been reported 23-26, the effect of EVO *in vivo* of TC cells was unclear. In this study, we showed that EVO exerts anticancer effects by inducing cell cycle arrest, inhibiting cell proliferation, promoting apoptosis and autophagy, and suppressing colony formation *in vitro*. To further evaluate its therapeutic efficacy and safety* in vivo*, a mouse xenograft model was established using subcutaneous injection of ARO cells into the right flank of nude mice, followed by oral administration daily of EVO with 5 or 15 mg/kg. Tumor growth was monitored every three days, along with bodyweight measurements. As shown in Figures 6A, treatment with EVO at 15 mg/kg significantly reduced tumor volume compared to the control group. Similarly, final tumor weight was significantly lower in the EVO-treated group (Figure 6B). To assess the safety profile of EVO, bodyweight changes and histopathological examinations of major organs were conducted. No significant differences in bodyweight were observed between the control and treatment groups (Data not shown), and hematoxylin and eosin (H&E) staining revealed no pathological alterations in liver or kidney tissues (Figure 6D). Additionally, no notable immune cell infiltration was detected (Figure 6D). Tumor apoptosis *in vivo* was further confirmed by TUNEL assay, which demonstrated that EVO induced significantly apoptosis* in vivo* (Figure 6C). Collectively, these findings suggest that EVO possesses strong antitumor efficacy against human ATC cells and is well tolerated *in vivo*.

## Discussion

Although representing only 2%-5% of thyroid cancers, anaplastic thyroid carcinoma (ATC) is one of the most aggressive and lethal human malignancies, with a median survival of merely 3-9 months 12. Its frequent presentation with distant metastases and resistance to conventional therapies highlights the urgent need for novel and effective treatment strategies.

Evodiamine (EVO), a bioactive alkaloid isolated from *Evodia rutaecarpa*, has demonstrated potent antitumor activity across various human cancers 20-22. Our previous work revealed that EVO exerts inhibitory effects on follicular thyroid carcinoma (FTC), and other studies have shown its efficacy against papillary (PTC) and anaplastic thyroid carcinoma through mechanisms involving proliferation suppression, cell cycle arrest, and apoptosis induction 10, 23-26. Although administration with EVO can induce G2/M arrest and apoptosis in human ATC cells has been demonstrated in this study and the previous reports 23-26. In the present study, we are the first to elevate that EVO induces autophagy and modulation of EVO-mediated autophagy attenuates caspase activation, indicating that autophagy plays a contributory role in EVO-induced apoptosis (Figure 4 and 5). Moreover, EVO effectively suppresses tumor growth *in vivo* without evident toxicity, supporting its potential as a safe and promising therapeutic agent for ATC (Figure 6).

In this study, we show that EVO significantly inhibits proliferation in ATC cells by inducing G2/M phase cell cycle arrest (Figure 1 and 2). This effect aligns with prior reports in other cancer models, where EVO disrupts G2/M progression via regulation of cyclins and cyclin-dependent kinases (CDKs) 10, 23, 42-44. Specifically, EVO treatment downregulated cyclin A, cdc2, and cdc25C, while upregulating p21 and cyclin B1, indicating a blockade of mitotic entry. These molecular alterations suggest that EVO-induced arrest results from inactivation of the cyclin B1-cdc2 complex and enhancement of p21-dependent checkpoint control—key pathways that suppress cell cycle progression in rapidly dividing cancer cells.

In addition to its antiproliferative effects, EVO was found to induce apoptosis via the intrinsic (mitochondrial) pathway in both ARO and SW579 cells, as evidenced by Annexin V/PI staining, caspase-9 and caspase-3 activation, PARP cleavage, and the lack of caspase-8 activation (Figure 3). This aligns with prior reports demonstrating that EVO triggers mitochondrial depolarization and cytochrome c release in other malignancies 45, 46. Furthermore, pretreatment with the pan-caspase inhibitor Z-VAD-FMK significantly attenuated EVO-induced caspase activation and apoptosis, confirming the caspase-dependent nature of this process (Figure 3E). These data reinforce the notion that EVO acts as a potent apoptotic inducer in ATC, a cancer subtype known for its resistance to conventional apoptosis-inducing therapies.

EVO has been shown to induce autophagy across various tumor types through multiple molecular mechanisms. In murine Lewis lung carcinoma cells, EVO promotes autophagosome formation, enhances LC3-I to LC3-II conversion, and upregulates autophagy-related genes including Atg4b, Atg5, and Atg7 47. Notably, inhibition of autophagy via 3‑MA augments EVO-induced apoptosis, demonstrating that autophagy functions as a cytoprotective mechanism in this context 47. Similarly, in human gastric cancer cells, EVO concurrently activates autophagy and apoptosis 48. EVO-induced autophagy, evidenced by increased Beclin‑1 expression and acidic vesicular organelle formation, contributes to cytotoxicity 48. However, blocking autophagy via 3‑MA partially rescues cell death, indicating a pro-death role of EVO mediated autophagy in gastric cancer cells 48. In the present study, the evidence demonstrated that blocking autophagy with 3-MA in EVO treated ATC cells could attenuate caspase activation (Figure 5), suggesting that EVO-induced autophagy might be the up-stream of apoptosis in ATC cells. Autophagy induction under EVO treatment plays a pro-death role in human ATC cells.

EVO elevates intracellular Ca²⁺, further stimulating JNK-mediated autophagy has been reported in glioblastomas cells 49. Additionally, EVO administration also modulates PI3K/Akt and MAPK/ERK signaling pathways in pancreatic cancer cells 50. Moreover, EVO also suppresses autophagy by inhibiting the phosphorylation of signal transducer and activator of transcription 3 (STAT3) in pancreatic cancer cells 50.

An important and novel finding of this study is that EVO induces autophagy in ATC cells, as shown by increased autophagosome formation, LC3-II accumulation, and sensitivity to autophagy inhibition by 3-MA (Figure 4). Interestingly, blocking autophagy not only suppressed LC3-II expression but also reduced caspase-3 activation and PARP cleavage, suggesting a functional interplay where autophagy acts upstream of apoptosis (Figure 5). While autophagy has a dual role in cancer, either promoting survival or contributing to cell death depending on context, our results support a pro-apoptotic role for autophagy in EVO-treated ATC cells. This is in line with finding in gastric cancers where autophagy-mediated caspase activation enhances EVO-induced cell death 48. These insights imply that targeting the autophagy-apoptosis axis may be a viable strategy to potentiate the therapeutic effects of EVO in ATC cells.

The *in vivo* xenograft model further confirmed EVO's potent anticancer effects in the present study (Figure 6). Treatment with EVO significantly reduced both tumor volume and weight in mice bearing ARO-derived tumors, without inducing noticeable toxicity (Figure 6A and 6B). Histopathological analysis revealed no damage to vital organs such as the liver and kidneys, and body weight remained stable throughout treatment (Figure 6D). Importantly, TUNEL assays confirmed increased apoptotic activity in tumor tissues following EVO treatment (Figure 6C). These findings are consistent with earlier studies showing that EVO suppresses tumor progression *in vivo* in prostate and pancreatic cancers while maintaining a favorable safety profile 50, 51. The absence of immune cell infiltration also suggests that EVO does not provoke local inflammation, further supporting its potential as a safe and effective therapeutic agent for advanced thyroid cancers.

Interestingly, EVO treatment has been shown to suppress IFN-γ-induced PD-L1 expression in non-small-cell lung cancer cells, reduce T-cell apoptosis, and enhance CD8⁺ T-cell infiltration *in vivo* in a Lewis lung carcinoma model 52, suggesting a potential anti-cancer strategy through combination therapy with EVO and anti-PD-1 mAb. Moreover, Zhou et al. reported that EVO combined with anti-PD-1 therapy significantly increased the proportions of intratumoral CD4⁺ and CD8⁺ T cells, as well as central memory T cells (TCMs) in the spleen, thereby demonstrating potent immunotherapeutic efficacy in triple-negative breast cancer 53. In human ATC, however, infiltration of exhausted CD8⁺ T cells and M2 macrophages is increased, whereas NK cells, B cells, and M1 macrophages are reduced 54. In addition, PD-L1 expression is frequently high in ATC cases 55. Although EVO treatment in the present study did not reveal changes in immune cell infiltration (Figure 6D), this limitation may be attributed to the use of a nude mouse model. Taken together, these findings warrant further investigation into whether EVO in combination with immunotherapy could provide enhanced therapeutic benefit for human ATC.

Taken together, our findings highlight EVO as a multifaceted anticancer agent capable of inducing G2/M arrest, activating caspase-dependent apoptosis, promoting autophagy, and suppressing tumor growth *in vivo*. Given the poor prognosis and limited treatment options for ATC, the ability of EVO to target multiple cellular pathways makes it a promising candidate for further development. Future studies should investigate the molecular crosstalk between autophagy and apoptosis in greater detail, explore synergistic combinations with existing chemotherapeutics or radiotherapy, and assess the effects of EVO in immune-competent models to determine its potential role in immunomodulation. Additionally, pharmacokinetic and pharmacodynamic profiling of EVO will be essential to advance its clinical applicability.

## Figures and Tables

**Table 1:** IC_50_ values of evodiamine in human anaplastic thyroid cancer cells.

	Time (h)	Cell line	
		ARO	SW579
Evodiamine (μM)	24	ND	ND
	48	2.5 ± 0.12	4.9 ± 0.11
	72	1.79 ± 0.05	2.0 ± 0.08
